# Data acquisition approaches for single cell proteomics

**DOI:** 10.1002/pmic.202400022

**Published:** 2024-08-01

**Authors:** Gautam Ghosh, Ariana E. Shannon, Brian C. Searle

**Affiliations:** ^1^ Ohio State Biochemistry Program The Ohio State University Columbus Ohio USA; ^2^ Pelotonia Institute for Immuno‐Oncology The Ohio State University Comprehensive Cancer Center Columbus Ohio USA; ^3^ Department of Biomedical Informatics The Ohio State University Medical Center Columbus Ohio USA

**Keywords:** data dependent acquisition, data independent acquisition, mass spectrometry, multiplex, proteomics, single cell

## Abstract

Single‐cell proteomics (SCP) aims to characterize the proteome of individual cells, providing insights into complex biological systems. It reveals subtle differences in distinct cellular populations that bulk proteome analysis may overlook, which is essential for understanding disease mechanisms and developing targeted therapies. Mass spectrometry (MS) methods in SCP allow the identification and quantification of thousands of proteins from individual cells. Two major challenges in SCP are the limited material in single‐cell samples necessitating highly sensitive analytical techniques and the efficient processing of samples, as each biological sample requires thousands of single cell measurements. This review discusses MS advancements to mitigate these challenges using data‐dependent acquisition (DDA) and data‐independent acquisition (DIA). Additionally, we examine the use of short liquid chromatography gradients and sample multiplexing methods that increase the sample throughput and scalability of SCP experiments. We believe these methods will pave the way for improving our understanding of cellular heterogeneity and its implications for systems biology.

**Abbreviations**
DDA: data‐dependent acquisition
DIA: data‐independent acquisition
LFQ: label‐free quantification
LIT: linear ion traps
SCoPE: single‐cell proteomics by mass spectrometry
SCP: single‐cell proteomics
TMT: tandem mass tags

## 1 INTRODUCTION

Single‐cell proteomics (SCP) is a growing field that aims to characterize the proteome of individual cells. The advent of SCP allows researchers to understand complex biological systems by offering insights into cellular development, disease progression, immune responses, and drug resistance [1]. Most biological systems consist of a variety of cells with distinct molecular and functional properties [2, 3, 4, 5, 6]. Characterizing differences in distinct cellular populations requires the analysis of individual cell proteomes, as bulk proteomes only capture an average measurement of all proteins in a system [7, 8, 9]. SCP can unveil subtle differences that bulk analysis might overlook, shedding light on heterogeneous cell populations and uncovering details essential for understanding disease mechanisms and developing targeted therapies. Researchers in the SCP field have developed various MS‐based methods that can be utilized to identify and quantify thousands of proteins from individual cells. This review focuses on the role of data‐independent acquisition‐based (DIA) methods in SCP, offering promising solutions for comprehensive analysis by enabling simultaneous quantification of multiple proteins across individual cells that can enhance both throughput and proteome coverage.

SCP studies can provide complementary results to single‐cell transcriptomics by offering insights into the abundance of proteins present within individual cells. By studying gene expression in single cells, transcriptomics reveals networks that regulate cellular processes and key pathways in multiple diseases [10, 11, 12, 13, 14, 15, 16], yet it does not fully capture the functional outcomes present in the proteome [17, 18]. SCP offers a direct assessment of the proteome of individual cells, which can provide crucial information on functional cellular processes while also potentially providing information on post‐translational modifications. The field of SCP is both newer and less established than single‐cell genomics and transcriptomics [11, 19], which presents challenges in method development and standardization. Unlike in sequencing nucleic acids, proteins cannot be readily amplified, which raises concerns about sample loss during preparation and handling, thus necessitating sensitive and efficient analytical techniques. Furthermore, protein abundances exhibit a dynamic range of at least seven orders of magnitude, varying from as low as one copy per cell to as high as ten million copies per cell, whereas the mRNA dynamic range covers only three orders of magnitude [20, 21]. These issues pose the key challenges in detecting and quantifying low‐abundance proteins accurately.

Due to the lack of amplification, optimized sample preparation is essential for sensitively measuring peptides and proteins with SCP. Low sample amounts can affect sensitivity through limited detection, while accuracy and reproducibility can suffer from increased measurement variability in sample preparation and acquisition [22]. Techniques such as micromanipulation [23, 24, 25], fluorescence‐activated cell sorting [26, 27, 28], and microfluidics [29, 30, 31] have been used for isolating individual cells. Recent technologies such as nanoPOTS [32, 33, 34], cellenONE [35, 36], and single‐cell printers [37, 38, 39] have further evolved single‐cell sample preparation. NanoPOTS and cellenONE are microfluidic platforms that condense all preparation steps into a single standardized procedure, minimizing both sample loss and contamination. Meanwhile, single‐cell printers precisely deposit individual cells in high‐throughput plates for preparation and analysis. Nanofluidics techniques like micropipettes have also been utilized as a straightforward and cost‐effective solution for isolating single cells [40]. Integrating these methods into SCP workflows has led to increased reproducibility, sensitivity, and throughput.

Following sample preparation, two global mass spectrometry (MS) acquisition methods are typically used for SCP: data‐dependent acquisition (DDA) [41] and DIA [42, 43]. DDA uses an onboard computer to select precursor ions for fragmentation based on their intensities, which can stochastically miss low‐abundance peptides, resulting in lower run‐to‐run reproducibility. In contrast, DIA fragments precursor ions in specified isolation windows regardless of intensity. To measure the mass range covering most peptide precursors, this method utilizes preselected, wide isolation windows, which typically fragment multiple precursor ions simultaneously to maximize coverage, while maintaining an efficient scan cycle time [44, 45]. One advantage of DIA is the measurement scheme is more consistent and reproducible compared to DDA. However, DIA requires more complex data analysis pipelines due to increased interference from the simultaneous fragmentation of multiple precursor ions.

While different acquisition methods can be utilized to optimize SCP experiments, achieving high‐throughput analysis to match the scale of single‐cell transcriptomics remains a significant challenge. Single‐cell transcriptomics studies have demonstrated the importance of analyzing large cell populations to capture cellular diversity. Similarly, SCP must overcome technical and logistical barriers to reach comparable scales to transcriptomics. Several modern DDA and DIA methods tackle throughput questions using multiplexing, faster methods, or cheaper instrumentation. Last, we discuss additional challenges of implementing MS and non‐MS proteomics methods when sampling a limited number of ions.

## 2 INCREASING SINGLE‐CELL SAMPLE THROUGHPUT

As a single‐cell benchmark, Svensson et al. analyzed over 1000 single‐cell transcriptomics studies and found that larger studies using over 100,000 cells analyzed an average of 3000 cells per sample to account for heterogeneity within the cellular population [46]. For example, the human single‐cell transcriptomics atlas [47] was collected using 483,152 cells from 55 tissue samples, with an average of around 3300 cells per individual tissue sample. In SCP, achieving experiment volumes comparable to single‐cell transcriptomics studies requires a substantial time investment. Two major strategies have been explored to analyze sufficiently large numbers of cells in SCP studies within an achievable timeframe with MS: implementing fast gradients and utilizing multiplexing techniques. Fast LC‐MS gradients use shorter chromatographic separation times, typically 15–30 min per sample, which significantly increase the throughput in SCP analyses [48, 49, 50]. Fast gradients enable rapid separation and analysis of peptides generated from single‐cell samples, leading to increased efficiency in data acquisition, while simultaneously concentrating peptides in narrow elution peaks that improve signal detection. Capillary electrophoresis can also be paired with MS (CE‐MS) [51, 52]. CE‐MS has been shown to be robust in SCP and low‐input analysis, where over a thousand protein groups can be identified using low nanogram quantities of samples. Finally, the use of faster mass analyzers further accelerates the analysis of peptides.

Sample multiplexing refers to the simultaneous analysis of multiple samples within the same MS experiment [53, 54]. Each sample is uniquely labeled or tagged, which allows the samples to be differentiated during data acquisition and analysis. This not only increases throughput but also improves sensitivity and reproducibility. For example, DDA attempts to make a single measurement of each precursor ion that achieves a specified intensity threshold. This limits the reproducibility across samples of low‐abundant peptide measurements in two ways: (1) peptides with intensities near the threshold may not consistently be sampled, and (2) the MS2 measurement typically does not occur at the chromatographic apex of the peptide elution. Multiplexing improves sensitivity because the intensity threshold can be achieved by the combination of multiplexed samples, rather than each sample individually.

Overall, multiplexing can enhance the efficiency, sensitivity, and throughput of single‐cell analyses. Multiplexing techniques, such as tandem mass tags (TMT) [55] and mTRAQ [56], offer different approaches for the simultaneous analysis of multiple samples in a single experiment. TMT utilizes isobaric tags that label peptides with unique MS2 reporter ions, while mTRAQ employs distinct nonisobaric tags that covalently attach to the peptides, resulting in a precursor mass shift that distinguishes them from one another. TMT multiplexing has been utilized in experiments with up to an 18‐plex capacity [57], while mTRAQ labels have been used to achieve 3‐plex analysis with plexDIA [58]. These methods increase workflow complexity but they can achieve greater efficiency compared to label‐free quantification (LFQ), which does not employ chemical labels for multiplexing.

Finally, cost efficiency is another factor that can affect SCP throughput. In particular, nominal‐resolution instruments like linear ion traps (LIT) can provide several benefits in SCP experiments due to their affordability, fast scanning speed, and high sensitivity. Unlike high‐resolution Orbitraps, which typically require a much higher ion count for a suitable MS1 or MS2 scan, LITs can achieve suitable scans with low ion counts [59, 60, 61]. LITs could offer rapid scanning speeds and high sensitivity, improving the detection of low‐abundance peptides in single‐cell samples. Despite their lower resolution compared to Orbitraps, LITs can also detect a comparable number of peptides from samples with input amounts of less than 10 ng [60]. This increased peptide detection efficiency can be attributed to the ability to achieve suitable scans with lower ion counts, allowing for higher scan rates with narrower precursor isolation windows. LIT instruments are also more cost‐effective, allowing labs to potentially acquire multiple units for parallel processing of samples, thus reducing experiment time and increasing overall throughput.

## 3 ISOBARIC TAGGING SCP METHODS WITH DDA

TMT has been integrally associated with SCP since the publication of the Single–Cell Proteomics by Mass Spectrometry (SCoPE) method, which leverages isobaric tag labeling both for multiplexing and for signal enhancement [32, 62, 64]. In low‐input proteomics experiments, successful utilization of TMT multiplexing has been demonstrated at capacities of up to 18‐plex [57]. TMT utilizes isobaric chemical tags that covalently bind to peptides, allowing for multiplexed quantification of proteins across multiple samples [55]. Isobaric tag methods label peptides using amine‐reactive reagents with the same molecular weight but produce unique MS2 reporter ions during peptide fragmentation that can be read as a quantitative “barcode.” Each TMT tag maintains the same precursor mass, therefore the signal of sequence‐specific fragment ions from multiple cells effectively stacks, which increases peptide identification rates. The use of a carrier channel, or one TMT label applied to 25–200 pooled cells, can dramatically simplify data analysis at the cost of lowering the quantitative dynamic range [64]. TMT also offers the flexibility to analyze a larger number of samples in a shorter time frame, making it a valuable tool for high‐throughput SCP analysis [65, 66].

**FIGURE 1:**
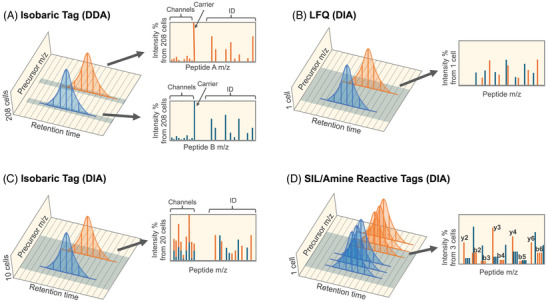
Comparison of MS spectra from different methods in single‐cell proteomics. (A) TMT‐DDA (Tandem Mass Tag with Data‐Dependent Acquisition) combines TMT labeling with data‐dependent acquisition, allowing for multiplexed quantification of proteins. In a TMT‐DDA MS2 spectrum, each peptide generates fragment ions. Alongside the fragment ions, specific reporter ions corresponding to each TMT channel are also observed. These reporter ions have unique masses that represent the different channels used for multiplexing. Meanwhile, the carrier ion helps increase sequence‐specific fragment ion intensity, which boosts the signal‐to‐noise ratio and enhances the detection of peptides during the analysis of the mass spectra. In this example, peptide signals have intensities of approximately 208 cells, where 200 come from the carrier channel combined with 8 single cells, as specified in the SCoPE method [62]. (B) LFQ‐DIA (Label‐Free Quantification with Data‐Independent Acquisition) relies on detecting and quantifying peptides between different single‐cell samples without using labels. Multiple peptide precursors can be fragmented simultaneously, typically resulting in highly complex MS2 spectra compared to DDA methods. (C) TMT‐DIA (Tandem Mass Tag with Data‐Independent Acquisition) multiplexes multiple samples within a single DIA experiment. One challenge of multiplexing both samples and peptides in the same measurement is that the same reporter ions in a TMT‐DIA spectrum can originate from multiple peptides, compromising their quantitative interpretation compared to TMT‐DDA. Without a carrier (as specified in Ctortecka et al. [77]), individual peptide signals would have a total intensity of 10 cells, whereas the MS2 signal can be the product of multiple peptides (e.g., 20 cells). (D) SIL‐DIA (Stable Isotope Labeling with Data‐Independent Acquisition) involves labeling proteins within cells with stable isotopes. In the MS2 spectra, N‐terminal b‐ions show distinct peaks corresponding to the different isotopic forms of the labeled peptides. These peaks are separated by the mass difference between the heavy isotopes. Meanwhile, C‐terminal y‐type ions may or may not be labeled depending on whether they contain a lysine, which results in y‐type ion peaks that are the combined intensities of all three isotopic variants.

Budnik et al. [62] applied TMT to multiplex quantification with the SCoPE2 workflow and measured approximately 200 cells a day [63]. In this approach, after digestion peptides from each cell are labeled with a TMT tag that produces a specific reporter ion that acts as a unique molecular signature to quantify peptides originating from that cell. TMT‐DDA, as used in the SCoPE‐MS method, combines the multiplexing capabilities of TMT labeling with data‐dependent acquisition (Figure 1A). However, the isobaric nature of the TMT tags introduces complexity in data interpretation, potentially leading to challenges in quantification accuracy and specificity [67]. Since all peptides contain the same reporter ion region, the quantification barcodes can be muddled if multiple peptides co‐elute in the same precursor isolation window. Averaging sample ratios tend to regress to the mean (e.g., 1:1) [68], which is why this phenomenon is conventionally called ratio compression, affecting as much as 20%–40% of peptides in DDA experiments [69, 70]. While MS3 is a powerful technique for ensuring reporter ion regions come from a single peptide [71], this approach reduces sensitivity because it dedicates additional time to measure the reporter ion region, which is problematic for SCP acquisition. Here, the use of fast‐scanning LIT mass analyzers to measure the reporter ion region can potentially be used in tribrid instruments [72] although the lower mass accuracy of LITs limits the potential isobaric tags to only nominal masses (e.g., TMT‐6plex). Finally, computational techniques [68, 69] can reduce ratio compression but can be sensitive to noisy data, which is exacerbated by the low dynamic range caused by using a carrier channel.

## 4 SCP USING LABEL‐FREE DIA

SCP using LFQ‐DIA detects peptides in single‐cell samples acquired with wide isolation windows [73]. Typically, multiple peptides are co‐fragmented in each MS2 so quantification is performed by the targeted extraction of co‐eluting fragment ions associated with a given peptide across a retention time window (Figure 1B). One major advantage of DIA over DDA is that each fragment ion is a potential quantitative peak, which allows software tools to pick and choose the best quantitative ions using transition refinement. Additionally, many fragment ions are more selective than precursor ions because they undergo quadrupole isolation in a precursor isolation window. The more narrow the isolation window, the less likely the fragment ions contain interference from other peptides. However, narrower isolation windows come at a cost in that more windows are required to cover the range where tryptic peptides typically occur. More windows means that a smaller percentage of the ion beam can be used for each window, lowering the total number of actual ions that go into each measurement [74].

LFQ‐DIA methods must be adapted in SCP studies to account for lower sample input. For example, Ye et al. developed an LFQ‐based SCP method that identifies over 5000 proteins and 40,000 peptides in single HeLa cells [48]. Fast LC gradients (15–30 min) coupled with DIA on an Astral mass spectrometer achieved a maximum capacity of 80 samples per day. Rather than the typical 2 m/z DIA window settings typically used on Astral‐based instruments [75], Ye et al. found it necessary to expand the precursor isolation windows to 4 m/z and a maximum of 6 ms ion accumulation times to achieve the highest number of detections. Both strategies effectively improve the efficiency of DIA methods by increasing the number of ions measured in ion‐poor situations. Similarly, Phlairaharn et al. modified LIT‐based DIA methods to use 10 m/z windows with 10 ms maximum accumulation [61], settings that differ widely from those optimized for bulk proteomics samples. To analyze SCP DIA data, the authors used the chromatogram library approach [76], which conducts gas‐phase fractionated DIA measurements of a cell pool to build a library for DIA analysis using as few as 40 cells, making it ideal for analyzing rare cell types, for example in the tumor microenvironment.

## 5 PITFALLS AND OPPORTUNITIES FOR COMBINING ISOBARIC TAGGING WITH DIA

TMT‐DIA (Figure 1C) combines the multiplexing strategy of TMT labeling with DIA to quantify single cells at a higher throughput [77, 78]. This approach takes advantage of the multiplexing provided by TMT labeling coupled with the ability to sample fragment ions across retention time, including at the peptide apex. However, TMT‐DIA also comes with challenges caused by multiplexing both samples and peptides in the same measurements, making it difficult to deconvolve which peptides produce which quantitative barcodes. While it is true that peptide signals are lower in SCP making it appear that interference is lower because it drops below the noise floor, the ratio of peptides within an experiment should remain consistent with bulk measurements. As such, the challenge of assigning quantitative data to peptides and samples is exacerbated by the fact that all peptides produce the same reporter ion barcodes regardless of whether those peptides were detected, and signals from undetected peptides are likely to be unaccounted for.

Additional work is needed to demonstrate if TMT‐DIA can manage ratio compression caused by coeluting precursors. Potential avenues for managing ratio compression include automatic data‐dependent MS3 acquisition for the most abundant ions [71, 72] or computational flagging of peptide ratios based on the level of estimated precursor impurity [68, 69]. A more practical solution may be to use complementary tandem mass tags (TMTc) that put the reporter ion barcode in a high‐mass region unique to each peptide mass [79]. With high‐plex TMT (greater than 6‐plex), reporter ion barcodes contain some channels that are separated by approximately 1 Da, but other channels differ only by as little as 0.006 Da. In the low‐mass range, a resolution of 60k can baseline separate these peaks, which is easily achievable with Orbitrap mass spectrometers. However, when this range is moved up to the range of peptide masses, resolution must be significantly higher [80], requiring over 100 msec transients even with super‐resolution techniques. While this approach can be challenging for bulk DIA methods that require faster MS2 rates to achieve typical precursor isolation windows, SCP DIA methods typically need longer ion accumulation times to collect enough ions to make a useful measurement. Consequently, future super‐resolution TMTc methods may make TMT‐DIA a practical and robust approach to multiplexing SCP measurements.

## 6 DIA USING STABLE ISOTOPE LABELS

Over the past quarter century, stable isotopic labeling procedures such as isotope‐coded affinity tag (ICAT) [81], Stable Isotope Labeling by Amino acids in Cell culture (SILAC) [82, 83], dimethyl labeling [84], and mTRAQ [56, 85] have been integral to the evolution of quantitative proteomics [86]. Briefly, ICAT labels cysteine residues with SIL modifications while dimethyl labeling and mTRAQ modify free amines. Conversely, SILAC incorporates isotopically labeled amino acids as part of protein synthesis in cells. Each method produces multiple precursor peaks for every peptide, where sample‐level quantification is encoded in the relative abundance of these peaks (Figure 1D). Since SILAC requires labeling in cell culture, it is difficult to use for SCP. However, mTRAQ has been used for SCP as part of PlexDIA [58, 87]. An additional innovation of PlexDIA is the use of only four extremely wide precursor isolation windows, enabling MS2 ion accumulation times for up to 300 ms. While this approach can increase sensitivity through long accumulation times, care must be taken when analyzing this data as fragments in each window could come from numerous peptides. Furthermore, since each window is between 120 and 580 m/z wide, peptides with similar sequences derived from paralogous genes can be easily overinterpreted if they fall within the same window.

**FIGURE 2:**
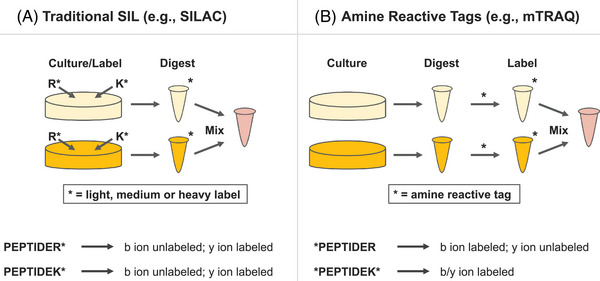
A comparison of traditional stable isotope labeling methods (like SILAC) and amine‐reactive tagging methods (like mTRAQ) for quantitative proteomics. (A) Stable isotope labeling first involves labeling cells in culture with stable isotopes of amino acids in three distinct forms: light (naturally occurring isotopic composition), medium (partially labeled), and heavy (fully labeled). Using this modality, peptides are labeled on the c‐terminus, which produces isomeric y‐type ions that indicate the quantity of each sample. (B) In contrast, mTRAQ utilizes nonisobaric tags to label peptides individually after cells are cultured, lysed, and digested. The labeled peptides are then combined for MS analysis. Here, peptides are labeled at free amines, shifting the masses of b‐type ions, which are typically more unstable and less useful for quantification. While some peptides that terminate in lysines will also have y‐type ion labels, typically quantification is performed at the precursor level, rather than the fragment level.

One potential problem with SIL labeling is that the approach pollutes the precursor space with multiple isotopic envelopes per peptide. Extra precursor peaks cause an overall reduction in detections using DDA due to competitive MS2 triggering [88]; however, this is controlled for in DIA experiments since precursor isolation windows are preprogrammed, rather than computer‐triggered. SILAC‐DIA is a well‐characterized procedure for multiplexing bulk cell samples grown with SIL lysine (K) and arginine (R) used for basic quantification and pulse‐chase analysis [89, 90, 91]. When digesting with trypsin, this approach produces peptides with C‐terminal labels and isotopically labeled y‐type ions, which can be used for fragment‐level quantification (Figure 2A). On the other hand, amine SIL labeling with mTRAQ and dimethyl labeling only guarantees labels on the N‐terminus, producing isotopically labeled b‐type ions (Figure 2B). Since b‐type ions are less stable than y‐type ions in beam‐type collision‐induced dissociation, quantification is typically performed using precursor ions only. This makes amine‐based SIL DIA quantification more akin to DDA in both accuracy and precision.

## 7 THE CHALLENGES OF SAMPLING A LIMITED NUMBER OF IONS

**FIGURE 3:**
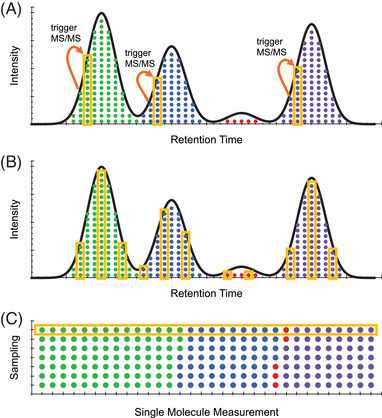
A comparison of DDA, DIA, and single‐molecule methods in SCP. (A) DDA attempts to make a single measurement of each precursor ion once the precursor intensity achieves a specific threshold. While this approach increases the number of detected peptides by avoiding measuring any given peptide multiple times, it limits the total amount of actual ions used to measure that peptide (orange boxes). Also, there is a stochastic chance that low abundance peaks never achieve the threshold and may be missed due to dynamic exclusion, which can limit sensitivity with low‐input samples. (B) DIA continuously samples a predefined mass range, which means that each peptide is sampled near its apex, leading to a higher likelihood of detecting low‐abundance peptides. (C) Single‐molecule measurements introduce the possibility of missing low‐abundance proteins because many highly abundant molecules must be sampled before the chance of observing low‐abundant molecules becomes a certainty.

Another significant challenge in SCP stems from the limited material available in single‐cell samples, where more time is needed to accumulate ions to generate a discernible signal. This limitation affects both DDA and DIA methods. DDA makes a single measurement of each precursor ion due to dynamic exclusion, which limits its sensitivity, especially when dealing with minimal sample material (Figure 3A). One approach to mitigate this challenge with DDA is to acquire fewer spectra by using longer ion injection times. For instance, The SCoPE2 method addresses this by employing a maximum of 100 ms ion accumulation times [63]. Alternatively, DIA continuously samples a predefined mass range, systematically fragmenting all ions within that range. This approach allows DIA to sample the same peptide multiple times, including the peak apex, making it inherently more sensitive (Figure 3B). Therefore, DIA is a viable option even for literal single‐cell samples, as it maximizes sensitivity by repeatedly sampling peptides, thus avoiding the need for extensive multiplexing strategies typically required by DDA. However, while DIA overcomes the sensitivity challenges posed by limited material, it introduces a trade‐off: requiring wider than normal isolation windows. Wider isolation windows increase the likelihood of interference from coeluting peptides and the chance of assigning or quantifying incorrect or interfered ions [92]. Despite these challenges, the benefits of DIA, including enhanced proteome coverage and reproducibility, make it a compelling choice for SCP studies.

Non‐MS‐based methods for SCP have also advanced significantly, offering alternate approaches to traditional MS‐based techniques by measuring single molecules. Proximity extension assays use pairs of antibodies coupled to unique DNA probes [93, 94, 95]. These probes come into proximity, ligate, and can be quantified via polymerase chain reaction (PCR) amplification [96]. Similar methods use DNA aptamers as binders [97, 98], allowing for immediate PCR readouts through sequencing. Nanopore‐based methods for protein sequencing are another promising option for single‐cell analysis [99, 100]. This procedure threads individual protein molecules through a nanopore and monitors changes in electrical current as each amino acid passes through the pore [101, 102]. Compared to MS‐based approaches, these methods offer unique advantages such as analyzing intact proteins, visualizing single molecules in real‐time, and directly quantifying proteins without the need for enzymatic digestion. However, measuring single molecules poses a challenge when analyzing the broad dynamic range of proteins in a single cell. Specifically, to detect even a single molecule per protein at the lower end of this range, single‐molecule quantification methods must generate tens of millions of measurements per cell (Figure 3C).

## 8 CONCLUSIONS

The development of DIA‐based SCP techniques is transforming analytical approaches to measure cell proteomes. DIA enables comprehensive proteomic analysis by fragmenting all precursor ions within a predefined mass range, which enhances the likelihood of detecting low‐abundance peptides, maximizing sensitivity. The reproducibility and efficiency of DIA also make it a compelling choice as it offers more consistent results compared to DDA. Additionally, DIA has the ability to provide unbiased peptide fragmentation, which enhances the accuracy of protein quantification, providing robust and precise measurements. These advantages highlight the use of DIA as a powerful tool in SCP. DIA methods have proven instrumental in overcoming limitations associated with traditional DDA approaches, offering increased proteome coverage and reproducibility while minimizing sample variability. The described DIA‐based methods, coupled with advancements in sample preparation workflows and MS instrumentation, have led to significant advancements in SCP research. Furthermore, the integration of DIA with multiplexing strategies, such as in plexDIA, has significantly increased sample throughput without significantly compromising quantification accuracy, sensitivity, and reproducibility, making high‐throughput SCP studies more accurate and efficient.

The primary limitation in SCP is achieving the desired throughput of over 3000 cells per sample, which is the average number of cells analyzed per sample in large‐scale single‐cell transcriptomics studies to capture cellular heterogeneity. Significant time investment is currently required to analyze 3000 cells per sample, with current methods like SCoPE taking around 30 days at approximately 100 cells per day using TMT for multiplexing. Other methods to achieve this goal include the utilization of faster gradients or lower‐cost approaches such as LIT‐based measurements, which would allow for multi‐instrument strategies to meet the necessary throughput to gain meaningful biological insights. Ultimately, a combination of all three techniques (multiplexing, faster gradients, and lower‐cost instrumentation) will likely be required.

In addition to addressing throughput challenges, it is also important to consider sampling statistics in SCP. The median protein copy count in a cell is around 50,000 compared to approximately 17 for transcripts [20], and this fundamental difference limits the quantitative accuracy of single‐cell transcriptomics due to the underlying number of molecules per transcript [103]. However, SCP's potential for increased sensitivity can overcome this limitation, allowing for more precise detection of quantitative differences. Given that proteomics reflects a closer approximation of real biological expression levels, SCP may achieve meaningful insights with fewer than the 3000 cells typically aimed for in large‐scale transcriptomics studies. We believe that advancements in DIA‐based SCP technologies will help increase our understanding of the role that cellular heterogeneity plays in systems biology, although further improvements in throughput will be essential for fully realizing its potential.

## CONFLICT OF INTEREST STATEMENT

Brian C. Searle is a founder and shareholder in Proteome Software, which operates in the field of proteomics.

## Data Availability

No new data was collected for this work.
